# Cholesterol lowering attenuates pressure overload-induced heart failure in mice with mild hypercholesterolemia

**DOI:** 10.18632/aging.102218

**Published:** 2019-09-04

**Authors:** Ilayaraja Muthuramu, Mudit Mishra, Joseph Pierre Aboumsallem, Andrey Postnov, Olivier Gheysens, Bart De Geest

**Affiliations:** 1 Centre for Molecular and Vascular Biology, Department of Cardiovascular Sciences, Catholic University of Leuven, Leuven 3000, Belgium; 2 Nuclear Medicine and Molecular Imaging, Department of Imaging and Pathology, Catholic University of Leuven, Leuven 3000, Belgium

**Keywords:** heart failure, transverse aortic constriction, hypercholesterolemia, gene therapy, oxidative stress

## Abstract

Epidemiological studies support a strong association between non-high-density lipoprotein cholesterol levels and heart failure incidence. The objective of the current study was to evaluate the effect of selective cholesterol lowering adeno-associated viral serotype 8 (AAV8)-mediated *low-density lipoprotein receptor (LDLr)* gene transfer on cardiac remodelling and myocardial oxidative stress following transverse aortic constriction (TAC) in female C57BL/6 LDLr^-/-^ mice with mild hypercholesterolemia. Cholesterol lowering gene transfer resulted in a 65.9% (p<0.0001) reduction of plasma cholesterol levels (51.2 ± 2.2 mg/dl) compared to controls (150 ± 7 mg/dl). Left ventricular wall area was 11.2% (p<0.05) lower in AAV8-LDLr TAC mice than in control TAC mice. In agreement, pro-hypertrophic myocardial proteins were potently decreased in AAV8-LDLr TAC mice. The degree of interstitial fibrosis and perivascular fibrosis was 31.0% (p<0.001) and 29.8% (p<0.001) lower, respectively, in AAV8-LDLr TAC mice compared to control TAC mice. These structural differences were associated with improved systolic and diastolic function and decreased lung congestion in AAV8-LDLr TAC mice compared to control TAC mice. Cholesterol lowering gene therapy counteracted myocardial oxidative stress and preserved the potential for myocardial fatty acid oxidation in TAC mice. In conclusion, cholesterol lowering gene therapy attenuates pressure overload-induced heart failure in mice with mild hypercholesterolemia.

## INTRODUCTION

Heart failure is the incapacity of the heart to eject blood forward at a sufficient cardiac output to meet the metabolic requirements of the body (termed forward failure) or the capacity to produce sufficient cardiac output only at the expense of pathologically elevated cardiac filling pressures (termed backward failure) or a combination of both. As the population ages, the public health impact of this syndrome will continue to increase [1].

The causal role of hypercholesterolemia in coronary artery disease is unequivocally established. However, plasma cholesterol lowering may also exert favourable effects on myocardial structure and heart function in the absence of atherosclerosis in the epicardial coronary arteries [2]. Increasing non-HDL cholesterol levels are an independent predictor of new-onset heart failure in multivariable models in Framingham Heart Study subjects that were free of coronary artery disease at the time of recruitment [3]. Detailed echocardiographic analysis of cardiac function in subjects with primary hypercholesterolemia without evidence of coronary artery disease revealed subclinical systolic and diastolic dysfunction [4]. The hypothesis that increased plasma cholesterol has a direct impact on the myocardium is also supported by experimental animal investigations. Adenoviral cholesterol lowering gene therapy improved systolic and diastolic function in mice with severe hypercholesterolemia [5]. Electrical remodelling of the heart [6] and systolic and diastolic dysfunction [7] were demonstrated in hypercholesterolemic rabbits *in vivo*. In addition, *ex vivo* cardiomyocyte studies showed a reduction of the peak rate of shortening and the peak rate of cardiomyocyte relaxation in cells isolated from hypercholesterolemic rabbits [8, 9]. However, the degree of hypercholesterolemia in these experimental rabbit investigations with plasma levels above 500 mg/dl undermines the relevance and the external validity of these observations. Moreover, clinical trials with proprotein convertase subtilisin/kexin type 9 inhibitors in human subjects with atherosclerotic vascular disease [10, 11] indicate that true normocholesterolemia and hypercholesterolemia in humans should be defined in terms of plasma cholesterol that are much lower than previously accepted.

We have recently reported the effect of cholesterol lowering adeno-associated viral serotype 8 (AAV8)-mediated *low-density lipoprotein receptor (LDLr)* gene therapy on cardiac function and remodelling in LDLr-knockout mice kept on a diet to induce marked hypercholesterolemia (0.2% (weight percentage) cholesterol 10% (volume percentage) coconut oil diet) [12]. AAV8-LDLr gene therapy improved both systolic and diastolic function in mice without pressure overload, whereas in mice with pressure overload following transverse aortic constriction (TAC), cholesterol lowering gene therapy counteracted structural and metabolic remodelling, improved cardiac function, and reduced lung congestion. Plasma cholesterol in this study was reduced from approximately 360 mg/dl to 60 mg/dl [12], an absolute cholesterol reduction of 300 mg/dl that is far higher than routinely observed in patients on hypolipidemic drugs. In addition, the hypercholesterolemic 0.2% cholesterol 10% coconut oil diet [13] contains the medium-chain fatty acid lauric acid (C12:0) as predominant fatty acid. A favourable impact of medium-chain fatty acids on the myocardium have been postulated [14]. Therefore, the effects of cholesterol lowering gene therapy in this prior study may have been moderated by the specific experimental study diet.

Considering the limitations of animal models of severe hypercholesterolemia in terms of clinical relevance, the goal of this investigation was to investigate the impact of selective cholesterol lowering in mice with mild hypercholesterolemia. We evaluated the effect of AAV8-LDLr gene transfer on cardiac remodelling, function, and metabolism, and on oxidative stress following TAC in female C57BL/6 LDLr^-/-^ mice with a baseline plasma cholesterol level of approximately 150 mg/dl. TAC initially leads to compensatory cardiac hypertrophy, but over time, the response to chronic pressure overload induces cardiac dysfunction with ensuing cardiac dilatation and heart failure.

## RESULTS

### Normalisation of plasma lipoprotein cholesterol levels following AAV8-LDLr gene transfer in mice with mild hypercholesterolemia

**Figure 1:**
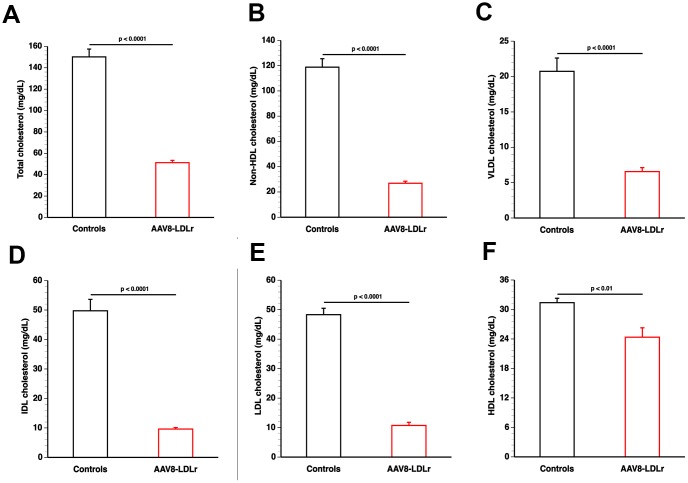
**AAV8-LDLr gene transfer normalizes lipoprotein cholesterol levels in C57BL/6 LDLr^-/-^ mice.** Bar graphs illustrating total cholesterol (**A**), non-HDL cholesterol (**B**), VLDL cholesterol (**C**), IDL cholesterol (**D**), LDL cholesterol (**E**), and HDL cholesterol (**F**) plasma levels in C57BL/6 LDLr^-/-^ mice at day 10 after AAV8-null gene transfer (controls) or AAV8-LDLr gene transfer. Lipoproteins were isolated by ultracentrifugation. Data are expressed as means ± SEM (n=7).

Total and non-HDL, VLDL, IDL, LDL, and HDL plasma cholesterol (mg/dl) at day 10 following AAV8-null (controls) or AAV8-LDLr gene transfer in female C57BL/6 LDLr^-/-^ mice fed standard chow diet are summarized in Figure 1. AAV8-LDLr gene transfer decreased plasma cholesterol levels by 65.9% (p<0.0001) compared to controls. Non-HDL, VLDL, IDL, and LDL cholesterol levels were reduced by 77.4% (p<0.0001), 68.4% (p<0.0001), 80.7% (p<0.0001), and 77.8% (p<0.0001) in AAV8-LDLr mice in comparison to control mice. Compared to controls, HDL cholesterol levels were 22.4% (p<0.01) lower in AAV8-LDLr mice (Figure 1). Plasma cholesterol levels in control mice and in AAV8-LDLr mice remained unchanged for the full duration of the experiment (data not shown).

### Decreased mortality in AAV8-LDLr TAC mice compared to control TAC mice

**Figure 2:**
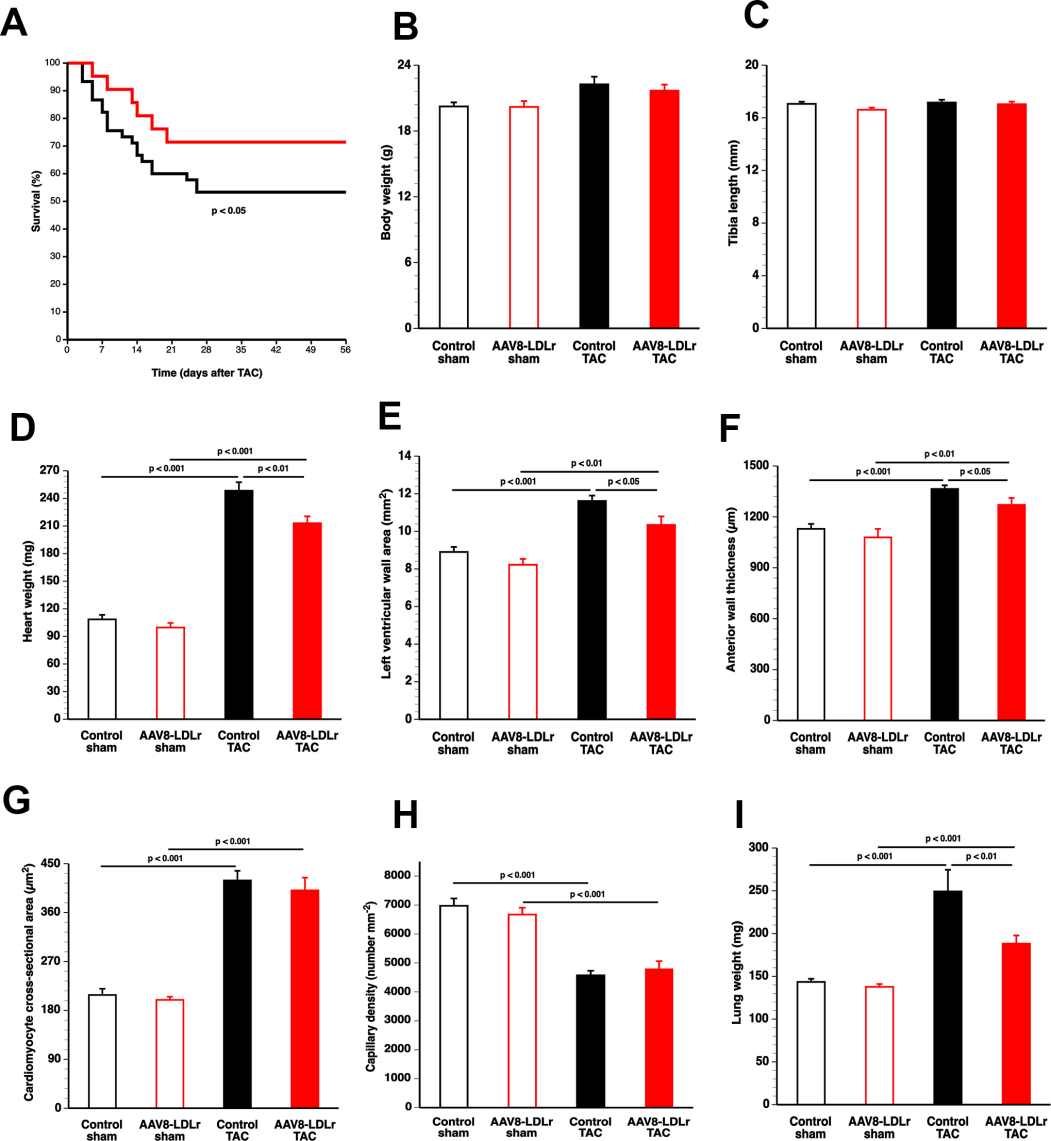
**Cholesterol lowering gene therapy improves survival, attenuates cardiac hypertrophy, and inhibits lung congestion after TAC.** Kaplan-Meier survival curves during an 8 weeks follow-up period comparing survival in control TAC mice (black) and AAV8-LDLr TAC mice (red) (**A**). Bar graphs illustrating body weight (**B**), tibia length (**C**), heart weight (**D**) in control sham (n=10), AAV8-LDLr sham (n=10), control TAC (n=9), and AAV8-LDLr TAC (n=10) mice 8 weeks after operation. Left ventricular wall area (**E**), anterior wall thickness (**F**) cardiomyocyte-cross-sectional area (**G**), and capillary density (**H**) quantified by morphometric and histological analysis in control sham (n=14), AAV8-LDLr sham (n=11), control TAC (n=25), and AAV8-LDLr TAC (n=11) mice 8 weeks after operation. Bar graph illustrating wet lung weight (**I**) in control sham (n=10), AAV8-LDLr sham (n=10), control TAC (n=9), and AAV8-LDLr TAC (n=10) mice 8 weeks after operation. Data are expressed as means ± SEM. Insets show a 4x magnification of the boxed region.

The TAC operation was executed at the age of 17 weeks. Mortality rate in AAV8-LDLr TAC mice during a follow-up period of 8 weeks was significantly lower than in control TAC mice (hazard ratio for mortality 0.423, 95% confidence interval 0.199 to 0.901) (Figure 2A). No mortality was observed in mice undergoing the sham procedure (data not shown).

### Cholesterol lowering AAV8-LDLr gene transfer potently inhibits cardiac hypertrophy and reduces lung congestion after TAC

**Figure 3:**
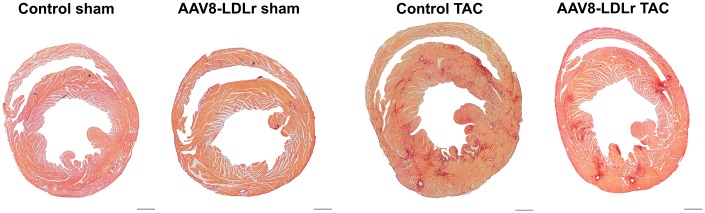
**Representative Sirius Red-stained cross-sections of sham hearts and TAC hearts at day 56 after operation.** Scale bar represents 1 mm.

**Figure 4:**
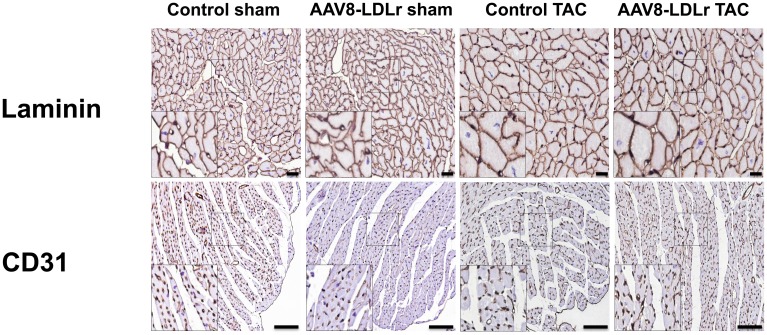
**Immunohistochemical analysis of the myocardium of sham mice and TAC mice at day 56 after operation.** Representative photomicrographs show laminin-stained cardiomyocytes and CD31-positive capillaries. Scale bar represents 50 μm.

Body weight (Figure 2B) and tibia length (Figure 2C) were not significantly different between sham and TAC groups. Cardiac weight was increased by 2.29-fold (p<0.001) and by 2.14-fold (p<0.001) in control TAC mice and AAV8-LDLr TAC mice, respectively, compared to respective sham groups (Figure 2D). Heart weight was 14.2% (p<0.01) lower in AAV8-LDLr TAC mice than in control TAC mice (Figure 2D), indicating a marked reduction of the degree of cardiac hypertrophy induced by cholesterol lowering gene therapy. Hypertrophy of the left ventricle was attenuated in AAV8-LDLr TAC mice as evidenced by an 11.2% (p<0.05) reduction of left ventricular wall area (Figure 2E) and a significant decrease of the anterior wall thickness (p<0.05) (Figure 2F). Figure 3 contains representative Sirius Red-stained cross-sections of sham hearts and TAC hearts, which clearly illustrate that cardiac hypertrophy following pressure overload was attenuated in AAV8-LDLr TAC mice compared to control TAC mice. At the microscopic level, a significant increase of cardiomyocyte cross-sectional area was observed in TAC groups compared to respective sham groups (Figure 2G). Myocardial vessel density was decreased by 34.4% (p<0.001) and by 28.1% (p<0.001) in control TAC mice and in AAV8-LDLr TAC mice, respectively, compared to respective sham groups (Figure 2H). Figure 4 comprises representative photomicrographs of laminin-stained cardiomyocytes and of CD31-positive capillaries, which illustrate cardiomyocyte hypertrophy and capillary rarefaction after TAC. Cholesterol lowering gene therapy diminished lung congestion as evidenced by the 24.5% (p<0.01) reduction of lung weight in AAV8-LDLr TAC mice compared to control TAC mice (Figure 2I). Atherosclerosis was undetectable in the left anterior descending coronary artery of sham and TAC mice. Taken together, these data indicate that cholesterol lowering AAV8-LDLr gene transfer in C57BL/6 LDLr^-/-^ mice with mild hypercholesterolemia inhibits ventricular hypertrophy and counteracts left ventricular failure after TAC.

### AAV8-LDLr gene therapy potently reduces pro-hypertrophic myocardial proteins in TAC mice

**Figure 5:**
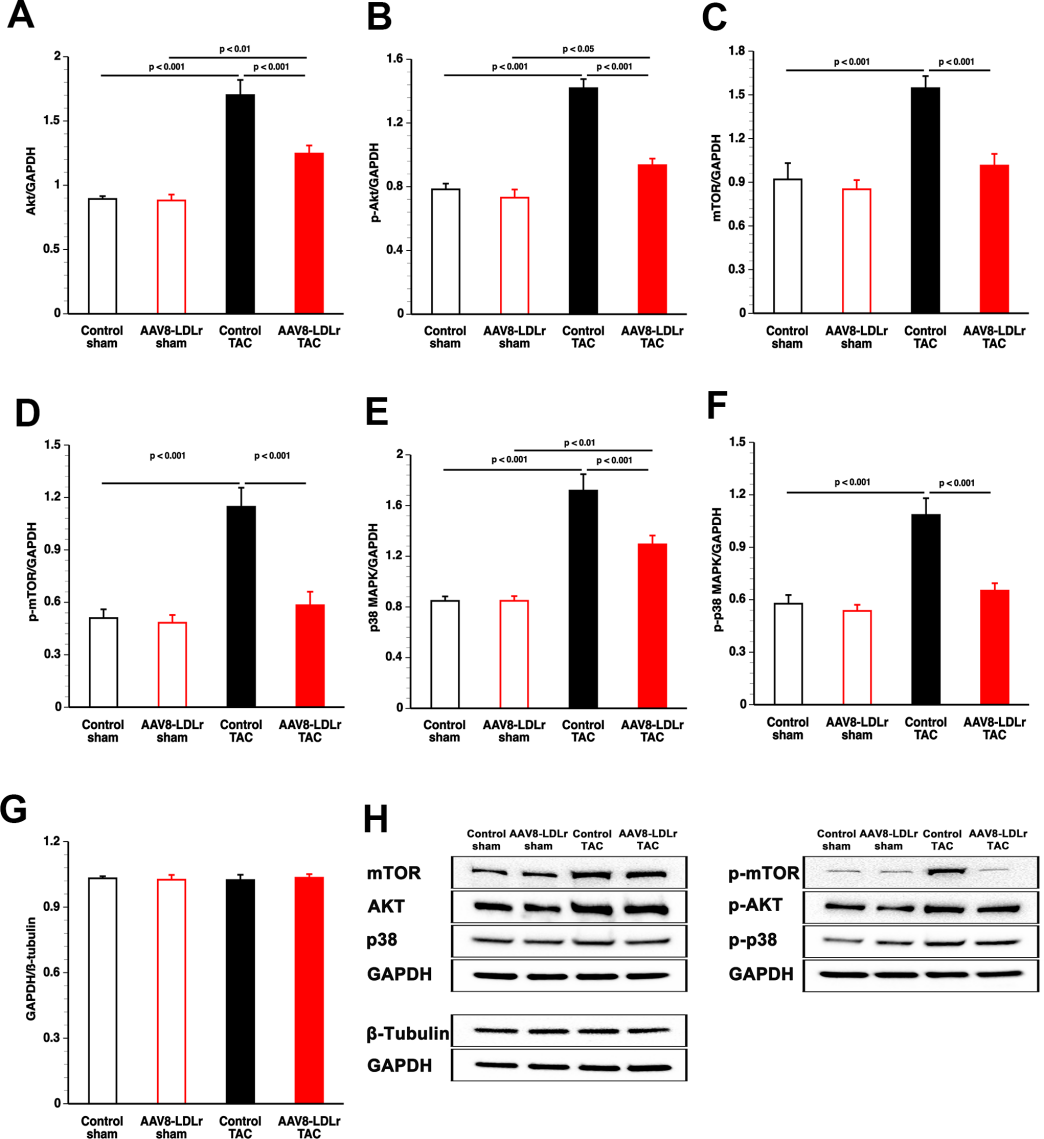
**Quantification of pro-hypertrophic myocardial proteins by western blot.** Bar graphs illustrating Akt (**A**), p-Akt (**B**), mTOR (**C**), p-mTOR (**D**), p38 MAPK (**E**), and p-p38 MAPK (**F**) protein levels quantified by western blot in the myocardium of control sham (n=8), AAV8-LDLr sham (n=8), control TAC (n=8), and AAV8-LDLr TAC (n=8) mice 8 weeks after operation. All protein levels were normalized to the glyceraldehyde-3-phosphate dehydrogenase (GAPDH) protein level. The GAPDH/ß-tubulin ratio is shown in panel g. Data are expressed as means ± SEM (n=8). Representative images of western blots are shown in panel h.

To investigate the anti-hypertrophic effects of cholesterol lowering gene therapy at the biochemical level, pro-hypertrophic myocardial proteins were quantified in TAC mice. Myocardial Akt (Figure 5A) and p-Akt (Figure 5B) protein levels were decreased by 26.7% (p<0.001) and by 34.1% (p<0.001), respectively, in AAV8-LDLr TAC mice compared to control TAC mice. Mammalian or mechanistic target of rapamycin (mTOR) (Figure 5C) and p-mTOR (Figure 5D) levels were reduced by 34.4% (p<0.001) and by 49.1% (p<0.001), respectively, in AAV8-LDLr TAC mice compared to control TAC mice. Myocardial protein levels of p38 mitogen-activated protein kinase (p38 MAPK) (Figure 5E) and of p-p38 MAPK (Figure 5F) were 24.7% (p<0.001) and 39.9% (p<0.001) lower, respectively, in AAV8-LDLr TAC mice than in control TAC mice. To verify that the housekeeping protein glyceraldehyde 3-phosphate dehydrogenase (GAPDH) is a reliable reference for normalizing protein levels, GAPDH and ß-tubulin protein expression levels were directly compared (Figure 5G). GAPDH/ß-tubulin ratios were nearly identical in the four different groups (Figure 5G). Representative western blot images are provided in Figure 5H.

### Cholesterol lowering decreases apoptosis and reduces interstitial and perivascular myocardial fibrosis in mice subjected to pressure overload

**Figure 6:**
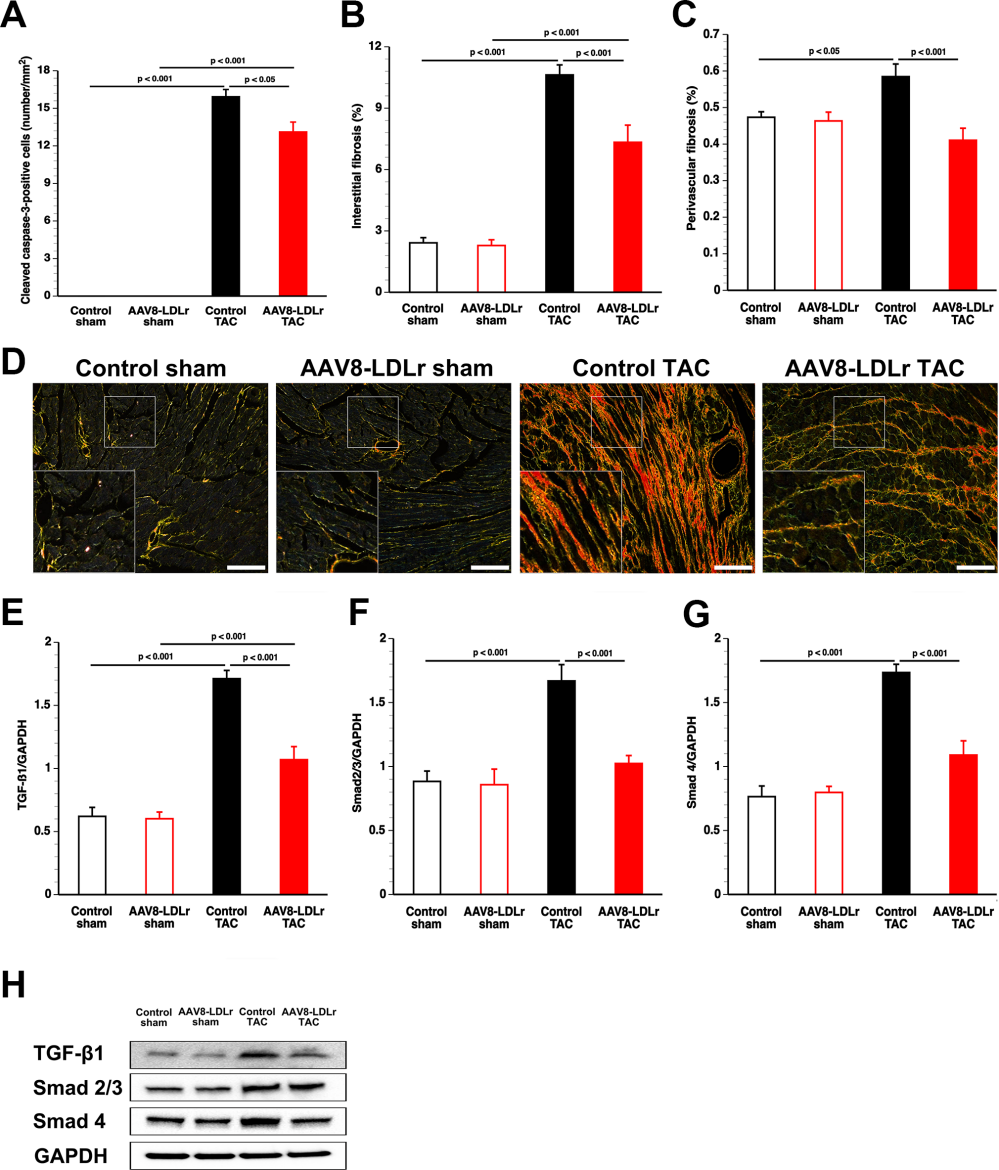
**AAV8-LDLr gene transfer significantly reduces interstitial fibrosis, perivascular fibrosis, and apoptosis after TAC.** Bar graphs illustrating the number of cleaved caspase-3 positive cells (**A**), the degree of interstitial fibrosis (**B**), and the degree of perivascular fibrosis (**C**) in control sham (n=14), AAV8-LDLr sham (n=11), control TAC (n=25), and AAV8-LDLr TAC (n=11) 8 weeks after operation. Representative photomicrographs showing Sirius Red-stained interstitial collagen viewed under polarized light (**D**). Scale bar represents 50 μm. Bar graphs illustrating the 25 kD isoform of TGF-ß1 (**E**), Smad2/3 (**F**), and Smad4 (**G**) myocardial protein levels quantified by western blot in the myocardium of control sham (n=8), AAV8-LDLr sham (n=8), control TAC (n=8), and AAV8-LDLr TAC (n=8) mice 8 weeks after operation. All protein levels were normalized to the glyceraldehyde-3-phosphate dehydrogenase (GAPDH) protein level. Data are expressed as means ± SEM. Representative images of western blots are shown in panel h. Insets show a 4x magnification of the boxed region.

Apoptosis in the myocardium of C57BL/6 LDLr^-/-^ mice was quantified using immunohistochemical staining of cleaved caspase-3. Cleaved caspase-3-positive cells were undetectable in myocardial tissue of sham mice (Figure 6A). Compared to control TAC mice, myocardial content of cleaved caspase-3-positive cells was decreased by 17.7% (p<0.05) in AAV8-LDLr TAC mice (Figure 6A). The extent of interstitial myocardial fibrosis (Figure 6B) and of perivascular myocardial fibrosis (Figure 6C) was reduced by 31.0% (p<0.001) and by 29.8% (p<0.001) lower, respectively, in AAV8-LDLr TAC mice, compared to control TAC mice. Figure 6D contains representative photomicrographs of Sirius Red-stained interstitial collagen viewed under polarized light illustrating reduced myocardial fibrosis following cholesterol lowering gene therapy.

The 25 kD isoform of transforming growth factor (TGF)-ß1 was reduced by 37.5% (p<0.001) in AAV8-LDLr TAC mice compared to control TAC mice (Figure 6E). Cholesterol lowering gene therapy in TAC mice decreased Smad2/3 (Figure 6F) and Smad4 (Figure 6G) by 38.7% (p<0.001) and by 37.2% (p<0.001), respectively. These data are illustrated by representative images of western blots in Figure 6H.

### Cholesterol lowering gene therapy in C57BL/6 LDLr^-/-^ mice with mild hypercholesterolemia ameliorates cardiac function in the presence of pressure overload

Systolic cardiac function and diastolic cardiac function were not significantly dissimilar in AAV8-LDLr sham mice than in control sham mice (Table 1). Systolic cardiac function in AAV8-LDLr TAC mice was enhanced compared to control TAC mice as evidenced by a 1.18-fold (p<0.05) elevation of the peak rate of isovolumetric contraction (dP/dt_max_). Cholesterol lowering gene therapy ameliorated diastolic function in TAC mice as evidenced by a 1.18-fold (p<0.05) increment of the absolute value of isovolumetric relaxation (dP/dt_min_) and a 15.9% (p<0.05) decrease of the time constant of isovolumetric relaxation (Table 1).

**Table 1:** Hemodynamic parameters in the left ventricle and in the aorta 8 weeks after sham operation or after TAC.

	**Control sham**	**AAV8-LDLr sham**	**Control TAC**	**AAV8-LDLr TAC**
**Number of mice**	**10**	**10**	**18**	**11**
**Left ventricle**				
Peak systolic pressure (mm Hg)	105 ± 3	100 ± 2	164 ± 5^§§§^	178 ± 4^§§§^
End-diastolic pressure (mm Hg)	3.40 ± 0.42	2.21 ± 0.36	2.02 ± 0.58	2.47 ± 0.18
dP/dt max (mm Hg/ms)	13.3 ± 0.6	13.4 ± 0.8	9.83 ± 0.42^§§§^	11.5 ± 0.3*
dP/dt min (mm Hg/ms)	-11.3 ± 0.4	-11.0 ± 0.3	-9.14 ± 0.49^§§^	-10.7 ± 0.3*
Tau (ms)	4.60 ± 0.20	4.33 ± 0.10	6.01 ± 0.25^§§§^	5.06 ± 0.18*
Heart rate (bpm)	597 ± 15	608 ± 9	593 ± 15	605 ± 5
**Aorta**				
Mean pressure (mm Hg)	83.2 ± 1.8	82.0 ± 1.6	98.7 ± 2.3^§§§^	105 ± 1^§§§^*
Peak systolic pressure (mm Hg)	103 ± 2	100 ± 3	162 ± 4^§§§^	176 ± 2^§§§^*
Peak diastolic pressure (mm Hg)	64.6 ± 2.8	63.4 ± 2.3	59.6 ± 2.8	61.7 ± 2.3

Sham operation or TAC was performed at the age of 17 weeks. Data are expressed as means ± SEM. ^§§^: p<0.01; ^§§§^: p<0.001 versus respective sham groups; *: p<0.05 versus control TAC.

### AAV8-LDLr gene therapy in TAC mice reduces pro-oxidative enzymes, increases anti-oxidant defense systems, and decreases nitro-oxidative stress in the myocardium

**Figure 7:**
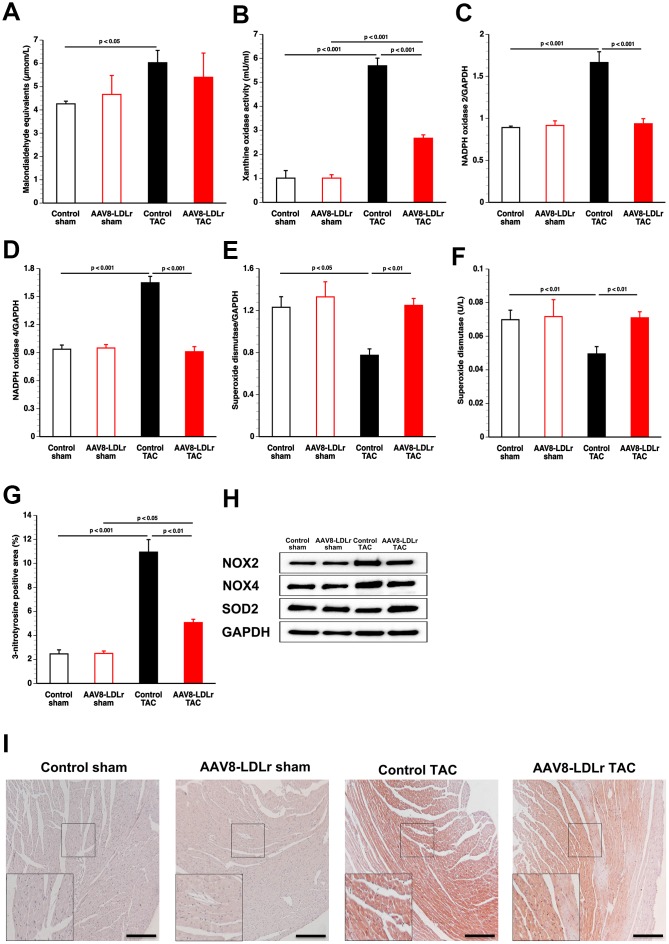
**Cholesterol lowering gene therapy in TAC mice reduces pro-oxidative enzymes, increases anti-oxidant defence systems, and decreases nitro-oxidative stress in the myocardium.** Bar graphs illustrating plasma TBARS expressed as plasma malondialdehyde equivalents (**A**), plasma xanthine oxidase activity (**B**), myocardial protein level of NADPH oxidase 2 (**C**), of NADPH oxidase 4 (**D**), and of superoxide dismutase (**E**), and plasma superoxide dismutase activity (**F**) (n=8 for each condition). Percentage of 3-nitrotyrosine-positive area in the myocardium in control sham (n=14), AAV8-LDLr sham (n=11), control TAC (n=25), and AAV8-LDLr TAC (n=11) mice 8 weeks after operation (**G**). Data are expressed as means ± SEM. Representative images of western blots are shown in panel h. Representative photomicrographs showing myocardial sections stained for 3-nitrotyrosine (**I**). Scale bar represents 100 μm. Insets show a 4x magnification of the boxed region.

Thiobarbituric acid reactive substances (TBARS) were 1.42-fold (p<0.05) increased in plasma of control TAC mice compared to control sham mice (Figure 7A). In contrast, no significant elevation of TBARS was discovered in AAV8-LDLr TAC mice compared to AAV8-LDLr sham mice. Xanthine oxidase activity in plasma was increased by 5.64-fold (p<0.001) and by 2.65-fold (p<0.001) in control TAC mice and in AAV8-LDLr TAC mice, respectively, compared to respective sham groups (Figure 7B). Xanthine oxidase activity was 53.1% (p<0.001) lower in AAV8-LDLr TAC mice than in control TAC mice. Myocardial NADPH oxidase 2 (Figure 7C) and NADPH oxidase 4 (Figure 7D) protein levels were reduced by 43.8% (p<0.001) and 44.8% (p<0.001), respectively, in AAV8-LDLr TAC mice compared to control TAC mice. Myocardial dismutase protein levels (Figure 7E) and plasma concentration of myocardial dismutase (Figure 7F) were 1.61-fold (p<0.01) and 1.43-fold (p<0.01) higher, respectively, in AAV8-LDLr TAC mice than in control TAC mice. Compared to respective sham groups, myocardial 3-nitrotyrosine-positive area (%) was increased by 4.45-fold (p<0.001) and by 2.03-fold (p<0.05) in control TAC mice and in AAV8-LDLr TAC mice, respectively. The 3-nitrotyrosine-positive area was 53.7% (p<0.01) lower in AAV8-LDLr TAC mice than in control TAC mice (Figure 7G), consistent with decreased nitro-oxidative stress.

### Cholesterol lowering AAV8-LDLr gene therapy counteracts metabolic remodelling induced by pressure overload

**Figure 8:**
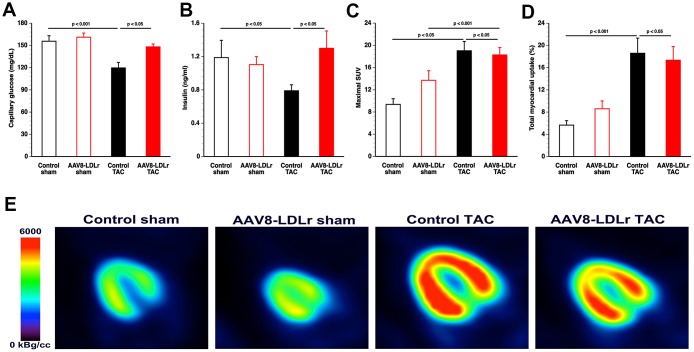
**AAV8-LDLr gene therapy counteracts metabolic remodelling.** Capillary glucose (**A**) and plasma insulin levels (**B**) 8 weeks after operation (n=10 for each group). Quantification of glucose uptake in the myocardium determined by micro-PET as shown by the maximal standardized uptake (SUV) value (**C**) and total myocardial uptake (% of injected dose) (**D**) 8 weeks after sham operation or after TAC (n=10-13 in each group). Representative micro-PET images illustrating the uptake of [^18^F]-FDG in the myocardium of sham mice and TAC mice at day 56 after operation are shown in panel (**E**).

Capillary glucose (Figure 8A) and plasma insulin (Figure 8B) levels were 23.1% (p<0.05) and 33.7% (p<0.05) lower, respectively, in control TAC mice than in control sham mice. Compared to control TAC mice, capillary glucose and plasma insulin levels were increased by 23.3% (p<0.05) and by 64.8% (p<0.05), respectively, in AAV8-LDLr TAC mice.

Myocardial uptake of glucose uptake was determined by micro-PET imaging using the tracer [^18^F]-fluorodeoxyglucose (FDG). Glucose uptake in the myocardium was significantly increased following TAC. The maximal standardized uptake value (SUV) (Figure 8C), the average SUV in voxels with a value above 50% of the maximal SUV (SUV 50%), and the average SUV in voxels with a value above 75% of the maximal SUV (SUV 75%) were significantly increased in control TAC mice (p<0.001) and in AAV8-LDLr TAC mice (p<0.05) compared to respective sham groups (Table 2). The total myocardial uptake of glucose was increased by 3.29-fold (p<0.001) and by 2.02-fold (p<0.05) in control TAC mice and in AAV8-LDLr TAC mice, respectively, compared to respective sham groups (Figure 8D). Glucose uptake in control TAC mice and in AAV8-LDLr TAC mice were highly similar. Differences in glucose uptake are illustrated by representative micro-PET images in Figure 8E.

**Table 2:** Quantification of glucose uptake in the myocardium determined by micro-PET 8 weeks after sham operation or after TAC.

	**Control chow**	**AAV8-LDLr sham**	**Control TAC**	**AAV8-LDLr TAC**
**Number of mice**	10	10	10	13
				
Maximal SUV	9.37 ± 1.03	13.7 ± 1.7°	19.0 ± 1.7^§§§^	18.3 ± 1.3^§^
				
SUV 50%	6.57 ± 0.77	9.61 ± 1.22°	12.9 ± 1.1^§§§^	12.6 ± 0.9^§^
Volume 50% (mm^3^)	99.6 ± 5.0	95.8 ± 4.5	141 ± 12^§^	138 ± 11^§^
				
SUV 75%	7.67 ± 0.89	11.4 ± 1.5°	15.7 ± 1.4^§§§^	15.2 ± 1.1^§^
Volume 50% (mm^3^)	43.3 ± 6.8	36.4 ± 2.8	40.7 ± 4.2	44.0 ± 3.9
				
% injected dose in myocardium (%)	5.65 ± 0.80	8.60 ± 1.41	18.6 ± 2.7^§§§^	17.3 ± 2.5^§^
				
SUV left quadriceps	0.598 ± 0.069	0.702 ± 0.048	0.621 ± 0.086	0.523 ± 0.047

Sham operation or TAC was performed at the age of 17 weeks. Sham operation or TAC was performed at the age of 17 weeks. SUV: standardized uptake value. SUV 50%: average SUV in voxels with a value above 50% of the maximal SUV. SUV 75%: average SUV in voxels with a value above 75% of the maximal SUV. Volume 50%: integrated volume of voxels with a value above 50% of the maximal SUV. Volume 75%: integrated volume of voxels with a value above 75% of the maximal SUV.

Data are expressed as means ± SEM. °: p<0.05 versus Control sham. ^§^: p<0.05; ^§§§^: p<0.001 versus respective sham groups.

To further evaluate the impact of cholesterol lowering on metabolic remodelling after TAC, metabolic myocardial proteins were quantified by western blot (Table 3). Cholesterol lowering AAV8-LDLr gene transfer in TAC mice decreased GLUT4 protein levels by 28.0% (p<0.001) and pyruvate dehydrogenase (PDH) levels by 30.0% (p<0.01) compared to control TAC mice. Myocardial protein levels of PDH kinase (PDHK), which leads to an inactivation of PDH, were decreased by 53.5% (p<0.01) in control TAC mice compared to control sham mice. PDHK levels were 2.10-fold (p<0.01) higher in AAV8-LDLr TAC mice than in control TAC mice (Table 3). Taken together, these data suggest that glucose oxidation may be lower in following cholesterol lowering AAV8-LDLr gene transfer in mice with pressure overload.

**Table 3:** Quantification of metabolic myocardial proteins by western blot.

	**Control sham**	**AAV8-LDLr sham**	**Control TAC**	**AAV8-LDLr TAC**
GLUT4/GAPDH	0.861 ± 0.067	0.814 ± 0.048	1.56 ± 0.10^§§§^	1.12 ± 0.04***
PDH/GAPDH	0.836 ± 0.054	0.907 ± 0.033	1.68 ± 0.08^§§§^	1.18 ± 0.11**
PDHK/GAPDH	0.904 ± 0.060	0.916 ± 0.040	0.420 ± 0.044^§§§^	0.884 ± 0.071**
AMPK/GAPDH	1.15 ± 0.08	1.15 ± 0.19	0.731 ± 0.035^§^	1.29 ± 0.08***
p-AMPK/GAPDH	0.675 ± 0.043	0.666 ± 0.029	0.387 ± 0.096^§^	0.848 ± 0.063***
ACC/GAPDH	0.891 ± 0.071	0.844 ± 0.051	1.55 ± 0.09^§§§^	1.25 ± 0.05^§§§^*
p-ACC/GAPDH	0.809 ± 0.022	0.828 ± 0.036	0.652 ± 0.075	0.968 ± 0.029***
PPAR-α/GAPDH	1.19 ± 0.07	1.31 ± 0.11	0.700 ± 0.037^§§^	1.24 ± 0.11***
CPT1B/GAPDH	1.07 ± 0.02	1.01 ± 0.04	0.647 ± 0.064^§§§^	1.03 ± 0.09***
LXR-α/GAPDH	1.01 ± 0.13	1.20 ± 0.09	0.651 ± 0.048^§^	1.49 ± 0.10***
LXR-ß/GAPDH	0.891 ± 0.076	0.881 ± 0.034	0.403 ± 0.083^§^	1.06 ± 0.04***

Sham operation or TAC was performed at the age of 17 weeks. Data are expressed as means ± SEM (n=8). ^§§^: p<0.01; ^§§§^

**Figure 9:**
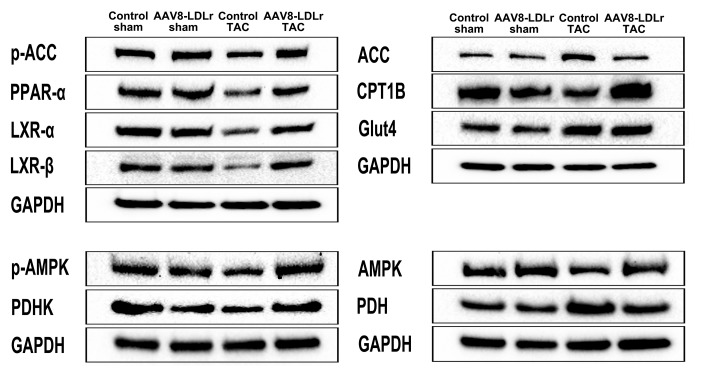
**Representative images of western blots of metabolic proteins.**

Myocardial protein levels of AMP-activated protein kinase (AMPK) and of p-AMPK were reduced by 36.2% (p<0.05) and by 42.7% (p<0.05), respectively, in control TAC mice compared to control sham mice (Table 3). Myocardial protein content of AMPK and p-AMPK was 1.76-fold (p<0.001) and 2.19-fold (p<0.001) higher, respectively, in AAV8-LDLr TAC mice than in control TAC mice. Acetyl-coenzyme A (acetyl-CoA) carboxylase (ACC) protein level in the myocardium was reduced by 19.4 % (p<0.05) in AAV8-LDLr TAC mice compared to control TAC mice. Myocardial protein content of p-ACC, which makes up the inactive form of the enzyme, was increased by 1.48-fold (p<0.001) in AAV8-LDLr TAC mice compared to control TAC mice. Peroxisome proliferator-activated receptor (PPAR)-α was 1.78-fold (p<0.001) higher in AAV8-LDLr TAC mice than in control TAC mice. Myocardial protein content of carnitine palmitoyltransferase IB (CPT1B) was increased by 1.60-fold (p<0.001) in AAV8-LDLr TAC mice compared to control TAC mice (Table 3). Taken together, data on ACC, p-ACC, PPAR-α, and CPT1B are consistent with a preserved capacity for myocardial fatty acid oxidation in AAV8-LDLr TAC mice and a reduced capacity for fatty acid oxidation in control TAC mice. Myocardial protein levels of liver X receptor (LXR)-α and of LXR-ß were 2.28-fold (p<0.001) and 2.63-fold (p<0.01) higher, respectively, in in AAV8-LDLr TAC mice than in control TAC mice. Figure 9 contains representative images of western blots illustrating the quantitative data of Table 3.

## DISCUSSION

The principal findings of the current study are that (1) cholesterol lowering gene therapy in C57BL/6 LDLr^-/-^ mice with only mild hypercholesterolemia significantly decreased mortality after TAC; (2) AAV8-LDLr gene transfer attenuated left ventricular hypertrophy, potently decreased interstitial myocardial fibrosis and perivascular myocardial fibrosis, and improved systolic and diastolic cardiac function after TAC; (3) cholesterol lowering AAV8-LDLr gene therapy counteracted heart failure as indicated by the pronounced reduction of wet lung weight compared to control TAC mice; (4) decreased myocardial nitro-oxidative stress resulting from reduced pro-oxidative enzymes and from enhanced anti-oxidant defence systems may be an important mediator of the observed favourable impact of cholesterol lowering gene therapy.; and (5) the preservation of the capacity for myocardial fatty acid oxidation may also have contributed to improvement of cardiac structure and function.

Whereas C57BL/6 LDLr^-/-^ mice kept on standard chow diet are hypercholesterolemic when normality is defined based on lipoprotein cholesterol levels in wild-type mice, the cholesterol level in control C57BL/6 LDLr^-/-^ mice would be considered to be consistent with normocholesterolemia in humans according to expert guidelines. However, clinical trials with proprotein convertase subtilisin/kexin type 9 inhibitors in patients with atherosclerotic vascular disease [10, 11] have shown that hypercholesterolemia in humans should be defined in terms of plasma cholesterol levels that are much lower than previously accepted. Therefore, cholesterol lowering in this study was induced in a range of values compatible with what can be achieved with contemporary hypolipidemic therapies. Moreover, the absolute cholesterol reduction of approximately 100 mg/dl in this experimental study is closer to what can be realistically achieved in clinical practice compared to the absolute cholesterol reduction of 300 mg/dl in our previous study [12]. Finally, the current study is a much more clear-cut demonstration of the impact of cholesterol lowering gene transfer on the progression of non-ischemic cardiomyopathy since the diet did not contain medium-chain fatty acids nor myristic acid, which were main components of the diet in the previous study [12]. Therefore, we can exclude that the impact of cholesterol lowering on myocardial structure and cardiac function was moderated by an unusual composition of the background diet.

Dyslipidemia in humans predicts left ventricular hypertrophy 20 years later independent of ischemic heart disease and valvular disease [15]. Cholesterol lowering induced by the AAV8-LDLr vector potently reduced cardiac hypertrophy after TAC. Myocardial Akt and p-Akt levels were significantly decreased following cholesterol lowering. Akt activation is pro-hypertrophic and prolonged activation of Akt may lead to heart failure [16–18]. Activated Akt can phosphorylate and activate mTOR or may inhibit PRAS40, which is an endogenous inhibitor of mTOR complex 1 (mTORC1) [19]. Reduced mTORC1 activity following AAV8-LDLr gene therapy in mice with pressure overload may be a key mediator of the pronounced impact on cardiac structure, function, and metabolism. Protein synthesis and cell growth are potentiated by mTORC1, whereas autophagy is inhibited [19, 20]. Moreover, increased mTORC1 leads to elevated glucose oxidation and decreased fatty acid oxidation [19, 20]. AAV8-LDLr gene transfer did not only reduce p-mTOR levels but also total mTOR levels, indicating regulation independent of Akt. Total myocardial mTOR levels have previously been shown to be increased in hypercholesterolemic swine [21] and hypercholesterolemic mice [12]. In addition, isolated hypercholesterolemia in rats suppressed basal cardiac autophagy and this decrease may have been the result of an activated mTOR pathway [22]. However, no changes in Akt, p-Akt, mTOR, or p-mTOR were observed following cholesterol lowering gene therapy in sham mice, indicating that isolated mild hypercholesterolemia in control sham mice is not sufficient to induce these alterations. Taken together, an interaction is observed between the metabolic phenotype and pressure overload that unmasks the effects of cholesterol lowering on these pro-hypertrophic pathways.

Cholesterol lowering gene therapy decreased p38 MAPK and p-p38 MAPK. Although p38 MAPK activity alone is insufficient to stimulate hypertrophy of cardiac myocytes *in vivo*, activation of p38 is more strictly linked to pathological hypertrophy than to physiological compensation [23]. Chronic p38 MAPK activation may diminish the force of contraction, has pro-apoptotic effects, and contributes to fibrosis [24]. Oxidized LDL activates p38 MAPK [25], which plays a critical role in inflammatory responses [26]. Activated p38 MAPK produces reactive oxygen species by up-regulating NADPH oxidase subunits [26]. In turn, the predominant forms of NADPH oxidases (2 and 4) cause reactive oxygen species-apoptosis signal-regulating kinase-dependent activation of p38 MAPK [26, 27]. In general, AAV8-LDLr gene therapy in TAC mice reduced pro-oxidative enzymes (xanthine oxidase, NAPDPH oxidase 2, NAPDPH oxidase 4) and increased anti-oxidant defence systems (superoxide dismutase). Reactive oxygen species in hypercholesterolemic animals can also be derived from dysfunctional mitochondria and from uncoupled nitric oxide synthase [28]. Nitro-oxidative stress was reduced in the myocardium. High levels of reactive oxygen species are involved in myocardial fibrosis, contractile dysfunction, and apoptosis [29, 30].

Myocardial uptake of glucose was not dissimilar between both TAC groups. This result should be interpreted cautiously since glucose and insulin levels were markedly decreased in control TAC mice compared to AAV8-LDLr TAC mice. In humans, a hyperinsulinemic-euglycemic clamp technique is applied during PET data acquisition. This is not feasible in mice from a logistic and operational point of view. Irrespective of the micro-PET results, it is likely that AAV8-LDLr gene therapy interfered with a shift from fatty acid to glucose as myocardial metabolic substrate in TAC mice [12]. Firstly, AAV8-LDLr gene transfer in mice with pressure overload reduced myocardial protein levels of PDH, which irreversibly catalyses the oxidative decarboxylation of pyruvate to generate acetyl-CoA, and augmented expression of PDHK, which deactivates PDH. Secondly, PPAR-α levels were significantly increased in AAV8-LDLr TAC mice compared to control TAC mice. The ligand-activated transcriptional factor PPAR-α is a major regulator of substrate metabolism. PPAR-α leads to the expression of CPT1B expression [31] and of PDHK [32]. Finally, data on myocardial protein content of ACC and of p-AAC, which is the deactivated form of the enzyme, indicate decreased ACC activity after cholesterol lowering gene therapy Consequently, malonyl-CoA-mediated inhibition of CPT1B [33], mediating transfer of fatty acids across the mitochondrial membrane, was less pronounced following AAV8-LDLr gene transfer in TAC mice.

From a clinical perspective, a clear distinction should be made between lipid lowering therapy for prevention of heart failure and initiation of lipid lowering therapy in patients with established heart failure. The major class of hypolipidemic drugs, namely 3-hydroxy-3-methylglutaryl-coenzyme A reductase inhibitors (statins), reduced heart failure incidence in both primary and secondary prevention trials [34] and decreased hospitalizations for heart failure during follow-up for 15 years of the West of Scotland Primary Prevention Study [35]. In clear contrast to these heart failure prevention studies, statins do not result in major favourable effects in patients with established cardiac failure [36, 37]. The results of this experimental murine study raise the hypothesis that more drastic cholesterol lowering may result in incremental effects in heart failure prevention.

### Limitations and future directions

The phenotype following TAC is modified by genetic background and by sex of the animals [38, 39]. It is reasonable to assume that there is no interaction between AAV8-LDLr gene transfer and genetic background/sex on the cardiovascular phenotype and heart failure in TAC mice. A second limitation is that cholesterol AAV8-LDLr lowering gene transfer was performed prior to TAC. Therefore, this experimental investigation is not equivalent to a clinical intervention trial in patients with established cardiac failure. Whether cholesterol lowering gene therapy ameliorates established non-ischemic cardiac failure induced by TAC represents an area for future research.

## CONCLUSIONS

AAV8-LDLr gene therapy in C57BL/6 LDLr^-/-^ mice with only a mild degree of hypercholesterolemia enhances survival, counteracts left ventricular hypertrophy, attenuates metabolic remodelling, and improves heart function in a model of non-ischemic cardiomyopathy. Reduced oxidative stress may be an important mediator of the observed effects.

## MATERIALS AND METHODS

### Construction, generation, and production of gene transfer vectors

Cholesterol lowering gene therapy was performed using an adeno-associated viral (AAV) serotype 8 vector containing a hepatocyte-specific expression cassette to induce expression of the murine low-density lipoprotein receptor (LDLr) (AAV8-LDLr). The expression cassette of this vector consists of the 1272 bp DC172 promoter, comprising an 890 bp α_1_-antitrypsin promoter fused together with 2 copies of the 160 bp α_1_-microglobulin enhancer, upstream of the human A-I 5’UTR containing the first intron (247 bp) followed by the murine LDLr cDNA sequence (2598 bp), and the rabbit ß-globin polyadenylation signal (127 bp) [12, 40, 41]. The control vector AAV8-null contains the transcriptional regulatory sequences but no insert. AAV vector production was performed as described [42].

### In vivo experiments

All experimental procedures in animals were performed in accordance with protocols approved by the Institutional Animal Care and Research Advisory Committee of the Catholic University of Leuven (Approval number: P154/2013). Female C57BL/6 LDLr^-/-^ mice, originally purchased from Jackson Laboratories (Bar Harbor, ME, USA), were fed standard chow diet (Sniff Spezialdiäten GMBH, Soest, Germany) following weaning. Gene transfer in C57BL/6 LDLr^-/-^ mice was performed at the age of 15 weeks by tail vein injection of 2 x 10^12^ genome copies/kg of AAV8-LDLr. Control mice were treated with an equivalent dose of AAV8-null. To induce pressure overload, transverse aortic constriction (TAC) was performed two weeks later [43]. TAC initially leads to compensatory hypertrophy of the heart, but over time, the response to chronic hemodynamic overload becomes maladaptive and results in cardiac dilatation and heart failure. Briefly, anaesthesia was performed with a single intraperitoneal injection of sodium pentobarbital (Nembutal^®^, Ceva Sante Animale, Brussels, Belgium) at a dose of 40-70 mg/kg. Mice were put in supine position and temperature was maintained at 37°C with a heating pad. A horizontal skin incision of 0.5 cm to 1 cm in length was made at the level of the suprasternal notch. A 2 mm to 3 mm longitudinal cut was performed in the proximal portion of the sternum and the thymus gland was retracted. This allowed visualization of the aortic arch under low-power magnification. A wire with a snare at the end was passed under the aorta between the origin of the right innominate artery and the left common carotid artery. A 7-0 silk suture (Ethicon, Johnson & Johnson, Livingston, Scotland) was snared with the wire and pulled back around the aorta. Subsequently, a bent 27-gauge needle (BD Microlance^®^, BD, Franklin Lakes, New Jersey) was placed next to the aortic arch and the suture was snugly tied around the needle and the aorta. Afterwards, the needle was quickly removed. The skin was closed and mice were allowed to recover on a warming pad until they were fully awake. The sham procedure was identical except that no constriction on the aorta was applied. Buprenorphine (Temgesic®) (Reckitt Benckiser Healthcare Ltd., Hull, UK) was administered at a dose of 0.1 mg/kg body weight subcutaneously for peri-operative pain relief. Postoperative analgesia was applied immediately following the intervention. Euthanasia of mice at the end of the experiment was performed by an intraperitoneal injection of sodium pentobarbital (200 mg/kg) and cervical dislocation.

Group assignment at the start of the study was performed at random. In the first experimental layer, mice were assigned for hemodynamic quantification and morphometric and histological analysis. The second experimental layer consisted of mice that did not undergo perfusion fixation and that were used for quantification of organ weights and for quantification of protein and mRNA levels. At the end of the study, data of all surviving mice were included in the analysis. Investigators who performed endpoint analyses were blinded to group allocation. Unblinding of animal numbers corresponding to specific allocation groups was performed at completion of measurements. All randomized mice were included in the analyses.

### In vivo hemodynamic measurements

Invasive hemodynamic measurements were performed 8 weeks after TAC or after sham operation as described [12, 43, 44]. Mice were anesthetized by intraperitoneal administration of 1.4 g/kg urethane (Sigma, Steinheim, Germany). Body temperature was maintained with a heating pad and monitored with a rectal probe. An incision in the right carotid artery was made with a 26-gauge needle between a distal and proximal non-occlusive ligation of the artery. A 1.0 French Millar pressure catheter (SPR-67/NR; Millar instruments, Houston, Texas, USA) was inserted and advanced to the left ventricle (LV). After stabilisation of the catheter, heart rate, maximal systolic LV pressure, minimal diastolic LV pressure, the peak rate of isovolumetric LV contraction (dP/dt_max_), and the peak rate of isovolumetric LV relaxation (dP/dt_min_) were measured. The end-diastolic LV pressure was calculated manually from the pressure in function of time curves. The time constant of isovolumetric LV pressure fall (tau) was calculated using the method of Weiss et al. [45]. Arterial blood pressure measurements were obtained after withdrawal of the catheter from the LV to the ascending aorta. Data were registered with Powerlab Bridge Amplifier and Chart Software (sampling rate 2000 Hz; ADInstruments Ltd, Oxford, United Kingdom).

### Blood sampling

Blood was obtained by puncture of the vena cava inferior at the end of the experiment just before euthanasia. Anticoagulation was performed with 0.1 volume of 136 mmol/L trisodium citrate and plasma was immediately isolated by centrifugation at 1100 x *g* for 10 minutes and stored at −80 °C.

### Plasma lipoprotein analysis

Mouse lipoproteins were separated by density gradient ultracentrifugation in a swing-out rotor as described before [46]. Fractions were stored at -20°C until analysis. Non-HDL cholesterol was determined as the sum of cholesterol within very low-density lipoproteins (VLDL) (0.95 < d < 1.006 g/ml), intermediate-density lipoproteins (IDL) (1.006 < d < 1.019 g/ml), and low-density lipoproteins (LDL) (1.019 < d < 1.05 g/ml) lipoprotein fractions. The cut-off value (d=1.05 g/ml) between LDL and high-density lipoproteins (HDL) for murine samples was chosen based on previous work by Camus, Chapman et al. [47]. Cholesterol in plasma and lipoprotein fractions was determined with commercially available enzymes (Roche Diagnostics, Basel, Switzerland). Precipath L (Roche Diagnostics) was used as a standard.

### Analysis of lipid peroxidation in plasma

Measurement of Thiobarbituric Acid Reactive Substances (TBARS) used for quantification of lipid peroxidation was performed according to the instructions of the manufacturer (Cayman Chemical, Ann Arbor, MI, USA).

### Quantification of myocardial protein levels by western blot

Myocardial tissue samples were isolated 56 days after sham operation or TAC and immediately frozen in liquid nitrogen and stored at -80°C. Tissues were placed in lysing matrix tubes (QBiogene/MP Biomedicals, Solon, OH, USA), mixed with 1 ml of protein extraction buffer containing 10 mM imidazole, 300 mM sucrose, 1 mM dithiotreitol, 1mM sodium metabisulfite, 25 mM sodium fluoride, 5 mM sodium ethylenediaminetetraacetic acid, 5 mM sodium pyrophosphate, 0.3 mM phenylmethylsulfonyl fluoride, and a protease inhibitor cocktail (Roche Diagnostics Belgium, Vilvoorde, Belgium), and homogenised in the FastPrep24 instrument (MP Biomedicals). Protein concentration was quantified using the Pierce BCA Protein Assay kit (Pierce Biotechnology Inc., Rockford, IL, USA). Equal amounts of proteins were separated on 4-20 % Tris-Glycine gradient gels (Bio-Rad Laboratories N.V., Temse, Belgium) and blotted onto polyvinylidene difluoride membranes (Bio-Rad Laboratories N.V.). Membranes were incubated with primary antibodies against Akt, phospho (p)-Akt (Ser/Thr), p38 MAPK, p-p38 MAPK (Thr180/Tyr182), mammalian or mechanistic target of rapamycin (mTOR), p-mTOR (Ser2481), acetyl-coenzyme A (acetyl-CoA) carboxylase (ACC), p-ACC (Ser79), AMP-activated protein kinase (AMPK)α, p-AMPKα (Thr172), Smad 2/3, Smad4, GLUT 4, pyruvate dehydrogenase (PDH), PDH kinase, transforming growth factor (TGF)-β1, ß-tubulin, glyceraldehyde 3-phosphate dehydrogenase (GAPDH) (all prior antibodies from Cell Signalling Technologies, Beverly, MA, USA), NADPH oxidase 2, NADPH oxidase 4, peroxisome proliferator-activated receptor (PPAR*-*α), carnitine palmitoyltransferase IB (CPT1B), liver X receptor (LXR)*-*α and LXR-ß (Abcam, Cambridge, UK). Protein expression was detected with Super signal west pico chemilumninescent reagents (Thermo Scientific, Rockford, IL, USA) and quantified using Image lab TM Analyzer software (Bio-Rad laboratories N.V.). All protein levels were normalized to the GAPDH protein level.

### Histological and morphometric analysis

Histological and morphometric analyses were executed as described [44]. After hemodynamic analysis, mice were perfused via the abdominal aorta with phosphate-buffered saline (PBS) and hearts were arrested in diastole by KCl (100 μL; 0.1 mol/L), followed by perfusion fixation with 1% paraformaldehyde in phosphate buffered saline. After dissection, hearts were post-fixated overnight in 1% paraformaldehyde, embedded in paraffin, and 6 μm thick cross-sections at 130 μm spaced intervals were made extending from the apex to the basal part of the left ventricle. Left ventricle (LV) remodelling was assessed by morphometric analysis on mosaic images of Sirius red-stained heart cross-sections using Axiovision 4.6 software (Zeiss, Zaventem, Belgium). Anterior wall thickness and septal wall thickness were determined. All geometric measurements were computed in a blinded fashion from representative tissue sections of 4 separate regions and the average value was used to represent that animal for statistical purposes [48, 49].

To measure collagen content in the interstitium, Sirius Red staining was performed as described by Junqueira et al. [50]. Sirius Red polarization microscopy on a Leica RBE microscope with KS300 software (Zeiss) was used to quantify thick tightly packed mature collagen fibers as orange-red birefringent and loosely packed less cross-linked and immature collagen fibers as yellow-green birefringent. Collagen positive area was normalized to the LV remote area and was expressed as percentage. Any perivascular fibrosis was excluded from this analysis. Perivascular fibrosis was quantified as the ratio of the fibrosis area surrounding the vessel to the total vessel area. Two mid-ventricular sections were studied per animal [5].

Cardiomyocyte hypertrophy was analysed on paraffin sections stained with rabbit anti-mouse laminin (Sigma; 1/50) by measuring the cardiomyocyte cross-sectional area (μm^2^) of at least 200 randomly selected cardiomyocytes in the LV myocardium. The capillary density in the myocardium was determined on CD31-stained sections using rat anti-mouse CD31 antibodies (BD; 1/500). Two mid-ventricular cross-sections were analysed per mouse [48, 49].

Immunostaining for 3-nitrotyrosine was performed with rabbit anti-nitrotyrosine antibodies (Merck Millipore, Overijse, Belgium; dilution 1/250).

Apoptosis was quantified on deparaffinised tissue sections using SignalStain^®^ cleaved caspase-3 IHC detection kit (Cell Signaling Technologies, Beverly, MA, USA), which utilizes a polyclonal rabbit antibody to the neoepitope peptide at the end of cleaved caspase-3 [43].

### Evaluation of cardiac glucose metabolism by micro-positron emission tomography (micro-PET)

Glucose uptake in the myocardium and in the skeletal muscle was quantified by micro-PET using [^18^]F-fluorodeoxyglucose (FDG) as a tracer (309 ± 22 μCi) [12]. Imaging was performed 60 min after tracer administration. Animals were anesthetized by inhalation of 2% isoflurane in 100% oxygen and underwent static imaging for 10 minutes on a micro-PET Focus 220 scanner (Concorde Microsystems, Knoxville, TN, USA). Images were reconstructed with ordered subset expectation maximization algorithm with 6 iterations (OSEM3D 6i) and analysed with PMOD v.3.4 (Pmod Technologies, Zurich, Switzerland). To exclude any effect of diurnal variability, micro-PET data acquisition was consistently performed within the same 2 hours’ time frame of the day. The standardized uptake value (SUV) is the ratio between the uptake in a specific volume of interest versus the average uptake in the whole body. The simultaneous quantification of skeletal SUVs was performed since myocardial glucose metabolism is not always parallel to skeletal and whole-body glucose metabolism [51].

### Quantification of superoxide dismutase concentration and xanthine oxidase activity in plasma

Superoxide dismutase plasma protein concentration was quantified using the Superoxide Dismutase Assay kit (Cayman Chemical, Ann Arbor, MI, USA). Xanthine oxidase activity were assayed using commercially available kits (Abcam, Cambridge, MA, USA) according to the manufacturer’s instructions.

### Statistical analysis

All data are expressed as means ± standard error of the means (SEM). Parameters between four groups were compared by one-way analysis of variance followed by Bonferroni multiple comparisons post-test for comparing sham groups, TAC groups, and sham versus respective TAC groups using GraphPad Instat (GraphPad Software, San Diego, USA). When indicated, a logarithmic transformation or a square root transformation or a non-parametric test was performed. Parameters between two groups were compared using Student’s t test. When indicated, a logarithmic transformation, a square root transformation, or a non-parametric Mann-Whitney test was performed. The assumption of Gaussian distribution was tested using the method Kolmogorov and Smirnov. Kaplan-Meier survival curves were analysed by log-rank test using Prism4 (GraphPad Software). A two-sided p-value of less than 0.05 was considered statistically significant.
